# Controlled Levofloxacin Release and Antibacterial Properties of β-Cyclodextrins-Grafted Polypropylene Mesh Devices for Hernia Repair

**DOI:** 10.3390/polym10050493

**Published:** 2018-05-03

**Authors:** Noor Sanbhal, Xiakeer Saitaer, Yan Li, Ying Mao, Ting Zou, Gang Sun, Lu Wang

**Affiliations:** 1 Key Laboratory of Textile Science and Technology of Ministry of Education, College of Textiles, Donghua University, 2999 North Renmin Road, Songjiang, Shanghai 201620, China; noorsanbhal@hotmail.com (N.S.); xaker2@163.com (X.S.); Maoying-dhu@163.com (Y.M.); zouting@dhu.eud.cn (T.Z.); gysun@ucdavis.edu (G.S.); 2 Department of Textile Engineering, Mehran University of Engineering and Technology Jamshoro, Sindh 76062, Pakistan; 3 College of Textiles and Fashion, Xingjiang University, 666 Sheng Li Road, Tian Shan, Wulumuqi 830046, China; 4 Division of Textiles and Clothing, University of California, Davis, CA 95616, USA

**Keywords:** drug release, levofloxacin antimicrobial, β-Cyclodextrin grafting, polypropylene, hernia repair

## Abstract

Mesh infection is a major complication of hernia repair. After knitted mesh implantation, bacteria can grow within textile structures causing infection. In this work, polypropylene (PP) mesh devices were two-step grafted with hexamethylene diisocyanate (HDI) and β–cyclodexrins (CD) and then loaded with suitable antimicrobial levofloxacin HCL for hernia mesh-infection prevention. First, oxygen plasma was able to create surface roughness, then HDI was successfully grafted onto PP fiber surfaces. Afterwards, CD was covalently grafted onto the HDI treated PP meshes, and levofloxacin HCL (LVFX) was loaded into the CD cavity of the modified meshes. The modified devices were evaluated for sustained antibiotic properties and drug-release profiles in a phosphate buffer, and sustained drug release was observed between interfaces of meshes and aqueous environment. The antibiotic-loaded PP mesh samples demonstrated sustained antibacterial properties for 7 and 10 days, respectively, against both Gram-negative and Gram-positive bacteria. The CD-captured levofloxacin HCL showed burst release after 6 h but later exhibited sustained release for the next 48 h. Among all samples, the modified mesh LVFX-6 was more stable and showed more sustained drug release and could be employed in future clinical applications.

## 1. Introduction

Hernia is a defect due to the rupture or protrusion of an organ at the weakest area of the human abdominal wall [1,2]. Synthetic meshes have been successfully implanted to reduce hernia recurrence. Among synthetic meshes, light-weight polypropylene (PP) meshes are most commonly used to support damaged tissues in operated abdominal wall hernias [3,4,5,6,7,8]. Nevertheless, a major complication of PP meshes after hernia repair is the development of infections caused by hernia repairs [8,9]. The main reasons for mesh infections include gastric perforation and the formation of fistulas due to the uneven surface topography of knitted meshes. Thus, mesh infection is a major cause of hernia failure, and it is difficult to treat it once bacteria biofilm formation develops on the surfaces of the hernia mesh devices [10,11,12]. Staphylococcus aureus (SA) is the major pathogenic bacterium causing severe infections [13].

Infections caused by hernia repairs can be treated using a prophylaxis technique with preoperative antibiotic treatments before mesh implantation. However, the prophylaxis technique does not work well on PP mesh materials due to the fact that PP is hydrophobic and does not absorb antibiotics [14,15]. Another way to reduce mesh infection is to supply heavy oral doses of antibiotics. However, taking such high amounts of antibiotics may cause severe side effects to the body of the patient. Thus, a controlled and durable antibiotic release system on the mesh materials could be a key technique to minimize mesh-related infections [16].

β-Cyclodextrins (CD) has been widely used in the preparations of medical devices to provide sustained antibiotic-release properties [17,18] due to its unique glucose ring structures and multiple hydroxyl groups with a hydrophobic inside cavity and hydrophilic outside [19,20]. CD can capture antimicrobial drugs and deliver them slowly for required durations [21]. The hydrophobic cavity helps CD to capture antimicrobial agents without forming covalent links, and the external hydroxyl groups can react with a wide range of chemicals such as hexamethylene diisocyanate [22,23]. Moreover, CD complexes with levofloxacin have been achieved previously [24] and levofloxacin is a promising antibacterial for wound-infection prevention, which was considered in this research [25].

Surface activation of PP fibers with cold oxygen plasma could be an appropriate surface treatment method to increase surface roughness and hydrophilicity of PP fibers without damaging their bulk properties [26] and can increase the reactivity of the PP fibers [27]. Therefore, cold oxygen plasma with low pressure was proposed to modify PP surfaces for further chemical grafting in this work.

Previously, cyclodextrin (CD)-based hydrogel, comprised of CD and hexamethylene diisocyanate (HDI), has been used for the preparation of antibacterial-coated polyester (Perietex) mesh for slower drug release [22]. However, polyester mesh is not suitable for implantation due to its multifilament braided nature, which results in different abdominal complications such as formation of fistulas, infection, and other postoperative complications [28].Our research group has previously used surface-grafted β-Cyclodextrin to capture and release water insoluble compounds such as triclosan in preparation of antimicrobial mesh materials. Triclosan was used as a model antibacterial to simulate the antibacterial properties of modified PP meshes. However, due to growing environmental and human safety concerns, the use of triclosan is limited in the design preparation of medical devices [29].

**Figure 1:**
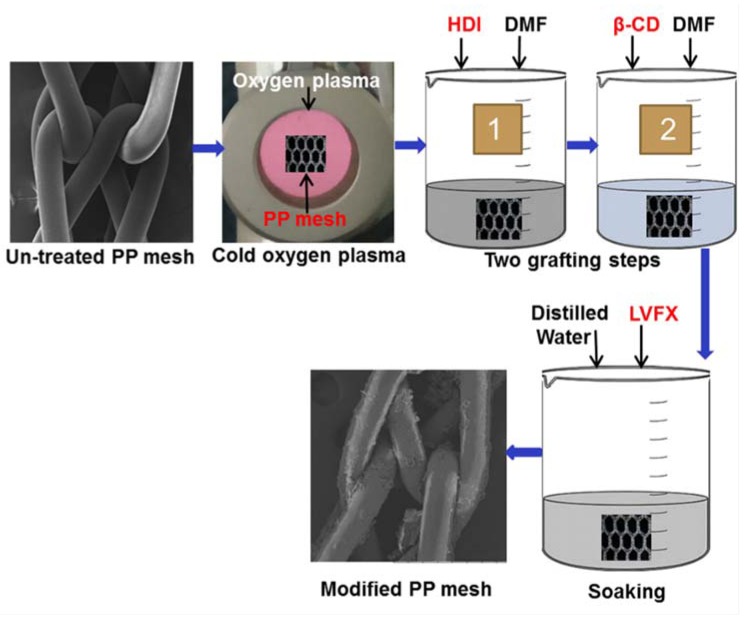
Illustrations of experimental design for preparation of antimicrobial PP meshes.

In this work, we used the antibiotic levofloxacin HCL (LVFX) in the CD-grafted PP mesh materials for the prevention of mesh infection. Oxygen plasma surface activated PP meshes were grafted with two-step-grafting HDI and then with CD. Afterwards, levofloxacin HCL was loaded into the grafted CD, as illustrated in Figure 1. Modified meshes were evaluated using controlled drug release by UV–Vis spectrophotometer. The antibacterial properties of the levofloxacin-loaded meshes were analyzed using the agar diffusion test method. Moreover, the characterization of the PP meshes, such as surface morphology, element analysis, and structural properties of CD-grafted meshes, were investigated using scanning electron microscope (SEM), Energy Despersive X-ray spectroscopy (EDX), and Fourier Transform Infrared Spectroscopy (FTIR). The results confirmed the structures and properties of the PP meshes. Moreover, the levofloxacin loaded PP meshes provided suitable controlled release of the antibiotics with sustained antibacterial properties for more than one week, which could be used in the prevention of mesh-related infections.

## 2. Materials and Methods

### 2.1. Materials

Light-weight (27 g/m^2^) and large-pore-size (3.5 mm × 2.5 mm) PP mesh was obtained from Nantong Newtec Textile and Chemical Fiber Co. LTD., Nantong, China. β-Cyclodextrin (CD) (≥97%) CAS: 7585-39-9 and hexamethylene diisocyanate (HDI) (≥98%) CAS: 822-06-0 was purchased from Sigma Aldrich, Shanghai, China. *N*-*N* dimethylformamide (DMF) Case No. 68-12-12 was purchased from Ling Feng Company, Shanghai, China. Levofloxacin hydrochloride (98%) was purchased from Energy Chemicals, Shanghai, China.

### 2.2. Methods

#### 2.2.1. Surface Functionalization of PP Meshes with Oxygen Plasma

Surface functionalization of PP mesh samples was done by using the low-pressure cold plasma treatment. The HD-300 cold plasma machine of radio frequency (13.56 MHz), power (500 W), and vacuum (300 cm × 300 cm) treatment chamber was used for the surface activation of meshes. All PP mesh devices of equal size (10 cm × 10 cm) were surface modified at optimized pressure (10 Pa), power (45 W), and time (300 s).

#### 2.2.2. Grafting HDI-CD onto PP Meshes in Two Steps

Oxygen-plasma-treated PP mesh fibers were grafted with β-Cyclodextrins using a two-step method. In the first step, the plasma-treated samples were reacted with HDI (2–6%). Afterwards, the PP-HDI-grafted samples were further reacted with CD (2–8%). A liquor ratio was set (mesh:solution) as 1:100 for both grafting processes. The maximum temperature and time were 75 °C and 60 min, respectively. Afterward, the surface-grafted PP samples were rinsed with warm distilled water (50 °C) to remove unreacted CD before drying up. The HDI-modified PP samples were named HDI-2 (2%), HDI-4 (4%), and HDI-6 (6%), and the CD-modified PP samples were named CD-2 (2%), CD-4 (4%), CD-6 (6%), and CD-8 (8%) according to the concentrations of HDI and CD in the solutions.

#### 2.2.3. Kinetics of Loading and Release of Levofloxacin HCL

The CD-modified PP samples (0.5 g) were soaked in a 50 mL aqueous solution containing 0.6% (0.3 g) levofloxacin HCL for 24 h. The levofloxacin HCL loaded samples were dried at 40 °C and named as LVFX-2, LVFX-4, LVFX-6, and LVFX-8, according to the different CD-grafted PP samples, respectively.

Moreover, the drug-release characteristics of the levofloxacin-HCL-loaded samples (LFVX–2 and LVFX-6) were measured to simulate their drug-release profiles. Meshes of 2 cm square were cut from LVFX-2 and LVFX-6. Samples were put in a centrifuge tube containing 8 mL PBS, and the mixtures were shaken at 70 rpm at 37 °C for the required durations. A buffer solution was prepared using disodium hydrogen phosphate dodecahydrate and sodium dihydrogen phosphate dihydrate. During absorption measurements, 1 mL of the mixture solution of each sample was extracted and a fresh 1 mL of buffer solution was added to the centrifugal tube. Absorption measurements were conducted using UV–Vis spectrophotometer (TU-1901 Beijing Purkinjie Co., Ltd., Beijing, China). Absorptions of all samples at 290 nm were measured. The accumulative drug-release rate (%) was determined as the ratio of released drug to the drug loaded onto the samples. Absorption values were recorded as the average values to calculate drug release (%).

## 3. Characterization 

### 3.1. SEM and EDX

The modified and untreated samples were coated (E–1045, Hitachi, Tokyo, Japan) with platinum (Pt) before testing on scanning electron microscope (SEM) by (Quanta 250, FEI^TM^, Hillsboro, OR, USA). However, for element analysis, an Energy Dispersive X-ray spectroscopy (EDX) oxford instrument unit (ISIS 300, Oxfordshire, UK) was connected with the SEM.

### 3.2. FTIR

All modified and control samples were analyzed using a Fourier Transform Infrared Spectroscopy (FTIR) Attenuated Total Reflection mode (ATR) (Nicolet 6700, Waltham, MA, USA). Samples were tested in the range of wave numbers 500–4000 cm^−1^ with resolution of 4.0 cm^−1^.

### 3.3. Antibacterial Activity Assessment

A qualitative analysis of antibacterial properties was assessed using an agar-diffusion test method, described in a recently published article [29]. A bacterial suspension of 400 µL (1 × 10^8^ CFU/mL) was evenly spread on agar plates using sterilized swabs. Escherichia coli ATCC25922 and Staphylococcus aureus ATCC 25923 (Shanghai, China) were selected for antibacterial activity of the modified meshes. All modified and control samples in the size of 1 cm × 1 cm square were placed on agar plates containing a suspension of bacteria. The agar plates containing the suspension and samples were incubated in an oven at 37 °C for 24 h. Afterward, the zone of inhibition was measured in four directions using a digital vernier caliper and reported as an average value. The following formula was used to calculate zone of inhibition:

*A* = (*B* − *D*)/2 (1)

where *A* = zone of inhibition, *B* = inhibition zone measured after incubation showing antibacterial activity, and *D* = inhibition zone before incubation.

Moreover, a serial plate-transfer test with agar plates was performed to confirm antibiotic release properties of the modified meshes. The objective of this test was to evaluate the antibacterial properties of the CD-grafted samples with reference to the antibacterial release time in days. The objective of the serial plate test was to evaluate release performance of levofloxacin according to the number of days. An agar plate with fresh bacteria suspension was changed after every 24 h, and samples were transferred onto a new agar plate containing equal colony-forming units (1 × 10^8^ CFU/mL) of Escherichia coli ATCC25922 or Staphylococcus aureus ATCC 25923, respectively. The serial plate-transfer test was continued until the antibacterial properties of the modified meshes were perceived.

## 4. Results and Discussion

### 4.1. Cyclodextrin Grafting and Levofloxacin HCL Loading

**Figure 2:**
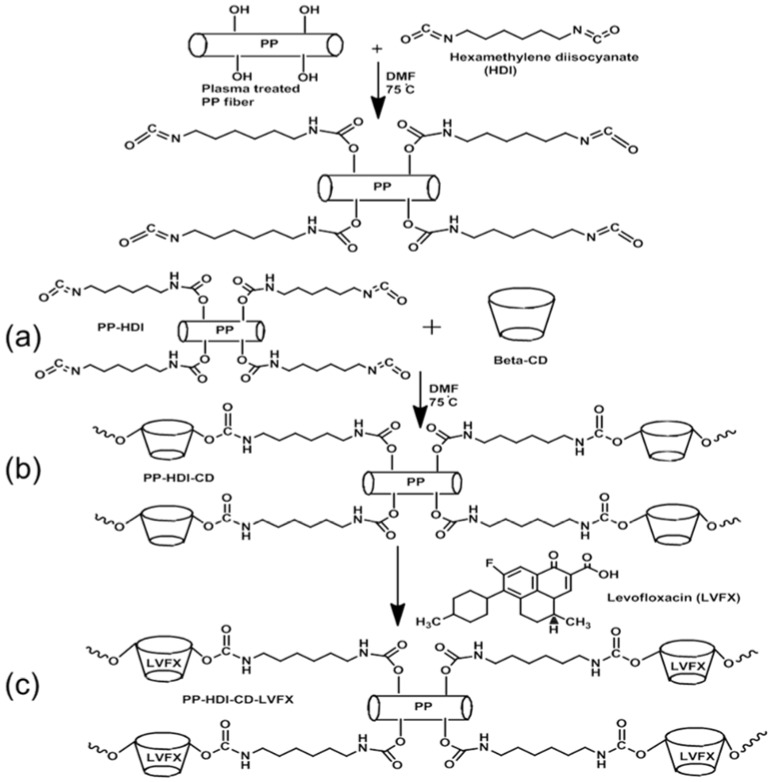
Two-step grafting (**a**) PP-HDI grafting (**b**) PP-HDI-CD grafting and (**c**) levofloxacin-loaded PP meshes.

As shown in Figure 2, CD was grafted onto PP meshes using two-step reactions. Oxygen plasma was able to activate PP meshes to create hydrophilic structures on the surfaces. Then, two grafting steps were carried out. In the first step (Figure 2a), HDI was successfully reacted with the plasma-treated meshes, and in the second step, the HDI-grafted PP mesh devices were further reacted with CD (Figure 2b). Thus, the grafted CD on the PP forms complexes with levofloxacin HCL. Afterward, levofloxacin HCL was successfully loaded into the CD-modified meshes (Figure 2c).

**Figure 3:**
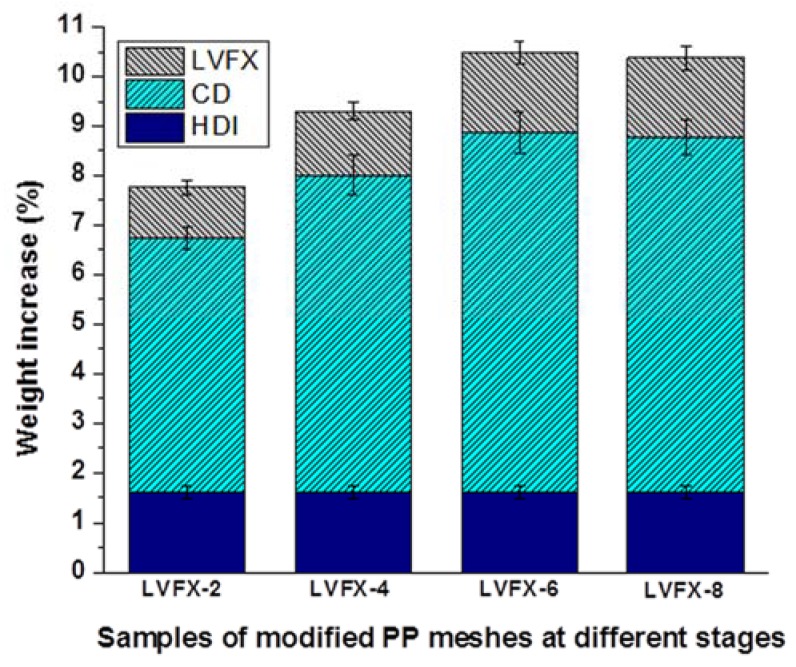
Weight-increase percentage of HDI, CD, and LVFX loaded samples. Data of each sample expressed as averages (*n* = 3).

The reaction time and temperature of the process were optimized and achieved at 60 min and 75 °C, respectively. Moreover, Figure 3 shows the weight-percentage increases of HDI, CD, and levofloxacin on the PP meshes, respectively. HDI-6 (6%) demonstrated better weight increases than others, and a thin layer of HDI coating was achieved on PP fibers. The corresponding weights of all plasma-treated meshes increased. However, the HDI-grafted meshes were immediately transferred to the CD solution without washing. Therefore, we were unable to measure the real weight increases of the HDI-grafted samples. All CD-incorporated samples were connected to the same concentration of HDI. Thus, the average weight increase of CDs for CD-2, CD-4, CD-6, and CD-8 were 5.128%, 6.40%, 7.271%, and 7.171%, respectively. However, after levofloxacin HCL loading, the average weight increases of LVFX-2, LVFX-4, LVFX-6, and LVFX-8 were 1.025%, 2.29%, 1.614%, and 1.60%, respectively. LVFX-6 had a greater weight increase compared with all other CD-grafted and levofloxacin-loaded samples. Therefore, LVFX-1 and LVFX-6 samples were chosen for further characterization and drug-release testing.

### 4.2. Surface Morphology of PP Meshes

**Figure 4:**
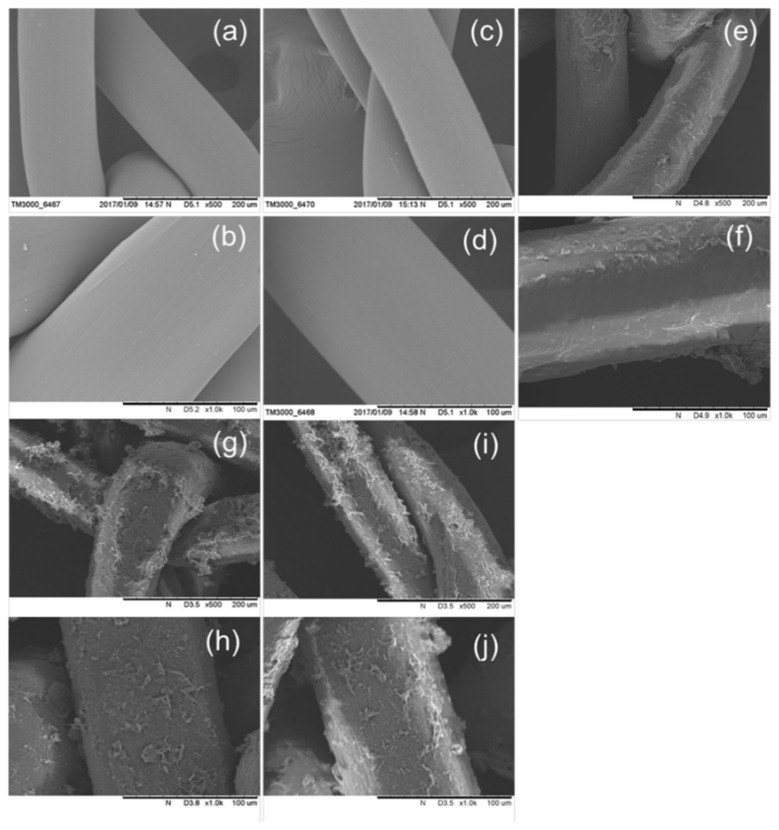
SEM images of: (**a**,**b**) PP control; (**c**,**d**) oxygen-plasma treated; (**e**,f) HDI-grafted HDI-6 (**g**,**h**) CD-grafted CD-6; and (**i**,**j**) levofloxacin-loaded (LVFX-6) (top row 500×, bottom row 1000×).

SEM images of the PP mesh samples are shown in Figure 4. The surfaces of the PP control before oxygen-plasma functionalization (Figure 4a,b) were very smooth. Nevertheless, after oxygen-plasma modification (Figure 4c,d), the PP mesh surfaces were slightly rougher. The meshes that were oxygen-plasma treated and grafted with HDI (Figure 4e,f) displayed a layer of HDI coating on the surfaces of the PP meshes. Additionally, the surfaces of the CD-grafted mesh samples can be observed (Figure 4g,h), which shows small beads and hills connected with spherical round-shaped fibers. It can be seen that (Figure 4i,j) after levofloxacin-loading, the CD-grafted meshes displayed similar results but with a little more swelling of the CD grafting onto the PP mesh fibers, showing that levofloxacin HCL loading has no effect on the structure of the fibers.

The results of the SEM images demonstrated that the surface grafting of HDI and CD on PP fibers was successful, confirming that low-pressure cold oxygen plasma is a suitable process to effectively incorporate CD onto PP meshes.

### 4.3. Characterization of HDI, CD Grafting, and Levofloxacin-Loaded Modified PP Mesh

**Figure 5:**
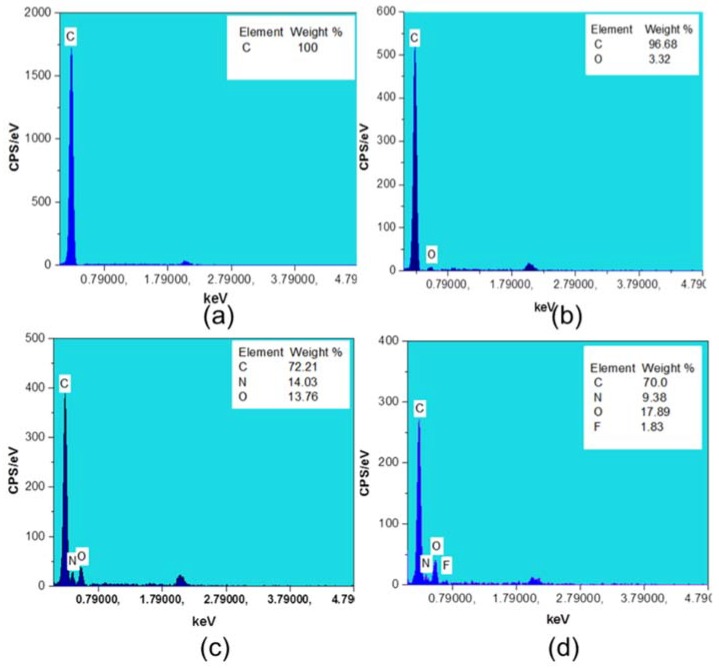
EDX spectra and element weight percentage (**a**) PP control; (**b**) oxygen-plasma treated (**c**) HDI-grafted HDI-6 (**d**) CD-grafted and levofloxacin-loaded LVFX-6.

Element analysis of untreated and surface-modified meshes was (Figure 5) conducted using Energy Dispersive X-ray spectroscopy (EDX).

It can be observed that the untreated PP control mesh (Figure 5a) sample displayed the presence of a carbon (C) atom within 0.5 keV. However, the plasma-treated mesh fibers showed carbon (C) and oxygen (O) atoms (Figure 5b) within 0.5 keV, which confirmed the modification of PP fibers with oxygen plasma. Moreover, Figure 5c shows one extra peak of nitrogen (N) on the modified PP, as well as oxygen (O) atom weight increase from 3.32% to 13.76%. All three peaks are within 0.5 keV, confirming the existence of HDI on the PP fiber surfaces. Moreover, cyclodextrins-grafted and levofloxacin-loaded LVFX-6 showed the existence of the same three elements—carbon (C), oxygen (O), and nitrogen (N)—at the same position within 0.5 keV but with slightly different peak heights. Furthermore, an additional peak of fluorine (F) atom showed up at 0.8 keV (Figure 5d), which confirmed the loading of levofloxacin HCL into the CD cavity. These results of the element analysis are in agreement with a recently published paper, except for the presence of the fluorine atom [29].

**Figure 6:**
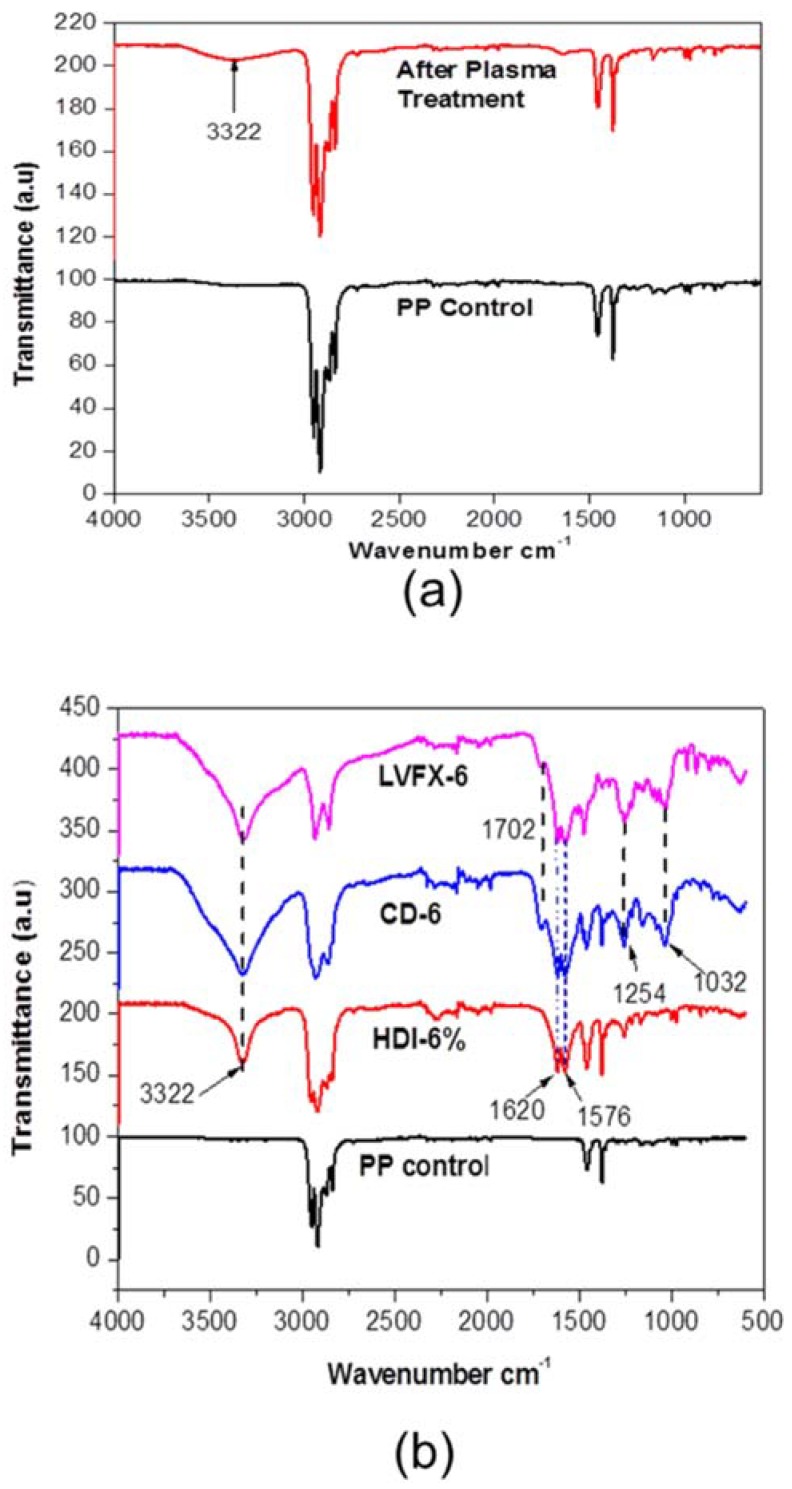
FTIR (ATR) spectra of; (**a**) PP control and oxygen-treated PP fibers (**b**) PP control, HDI-grafted, CD-grafted CD-6, and levofloxacin-loaded LVFX.

FTIR spectra of PP control, HDI, and CD-grafted mesh samples at different reaction stages were observed (Figure 6). The untreated PP showed absorbance bands at 2950 cm^−1^, 2916 cm^−1^, 1452 cm^−1^, and 1376 cm^−1^ for (va CH_3_), (va CH_2_), (δCH_3_), and (δCH_3_), respectively [30,31]. It can be observed that plasma-treated meshes showed absorbance bands at 3322 cm^−1^ (Figure 6a), and OH groups on the PP fibers were detected due to the surface activation with oxygen plasma. However, all other HDI- and CD-grafted samples displayed more absorbance peaks (Figure 6b) at 3322 cm^−1^, 1620 cm^−1^, and 1576 cm^−1^, showing hydroxyl (OH), amide I (ν C=O), and amide II (δNH) formations, respectively. It can be observed that the CD-grafted mesh samples maintained all peaks of HDI-grafted meshes and also showed absorbance peaks at 1701 cm^−1^ and 1254 cm^−1^ due to the formation of carbonyl groups (ν C=O) and C–H (ν CH), respectively. However, an absorbance peak at 1030 cm^−1^ was observed, which shows the C–O group of CD, and is consistent with the literature [29,32]. The absorbance bands of amide I at 1620 cm^−1^ and amide II at 1576 cm^−1^ confirmed CD-grafted samples and the formation of urethane, consistent with the literature as well [20,22]. Moreover, we did not find any absorbance peak related to the levofloxacin HCL probably due to the fact that levofloxacin HCL was captured by CD cavity.

### 4.4. Antibacterial Activity of Modified PP Meshes

**Figure 7:**
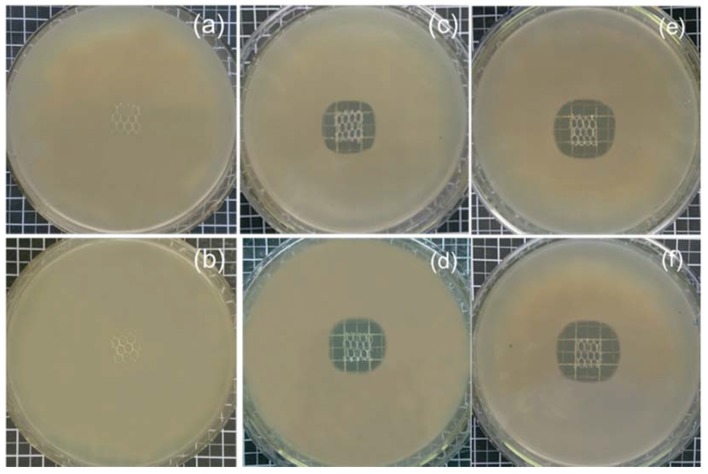
Antibacterial activity (**a**,**b**) PP control without modification, (**c**,**d**) LVFX-2, (**e**,**f**) LVFX-6, Top row *S. aureus* and bottom row *E. coli.*

The antibacterial performance of PP control and modified meshes was evaluated using an agar-diffusion test method as a qualitative analysis. Figure 7 shows control PP and the modified LVFX-2 and LVFX-6 meshes samples. The PP control and CD-grafted without levofloxacin HCL mesh samples did not show any antibacterial activity. However, the PP-grafted and levofloxacin-HCL-loaded mesh samples displayed suitable inhibition-zone diameters. The LVFX-2 showed average inhibition-zone diameters of 5.3 mm and 5.7 mm for *S. aureus* and *E. coli*, respectively. LVFX-6 showed increased inhibition-zone diameters of 6.7 mm and 6.9 mm for *S. aureus* and *E. coli*, respectively.

**Figure 8:**
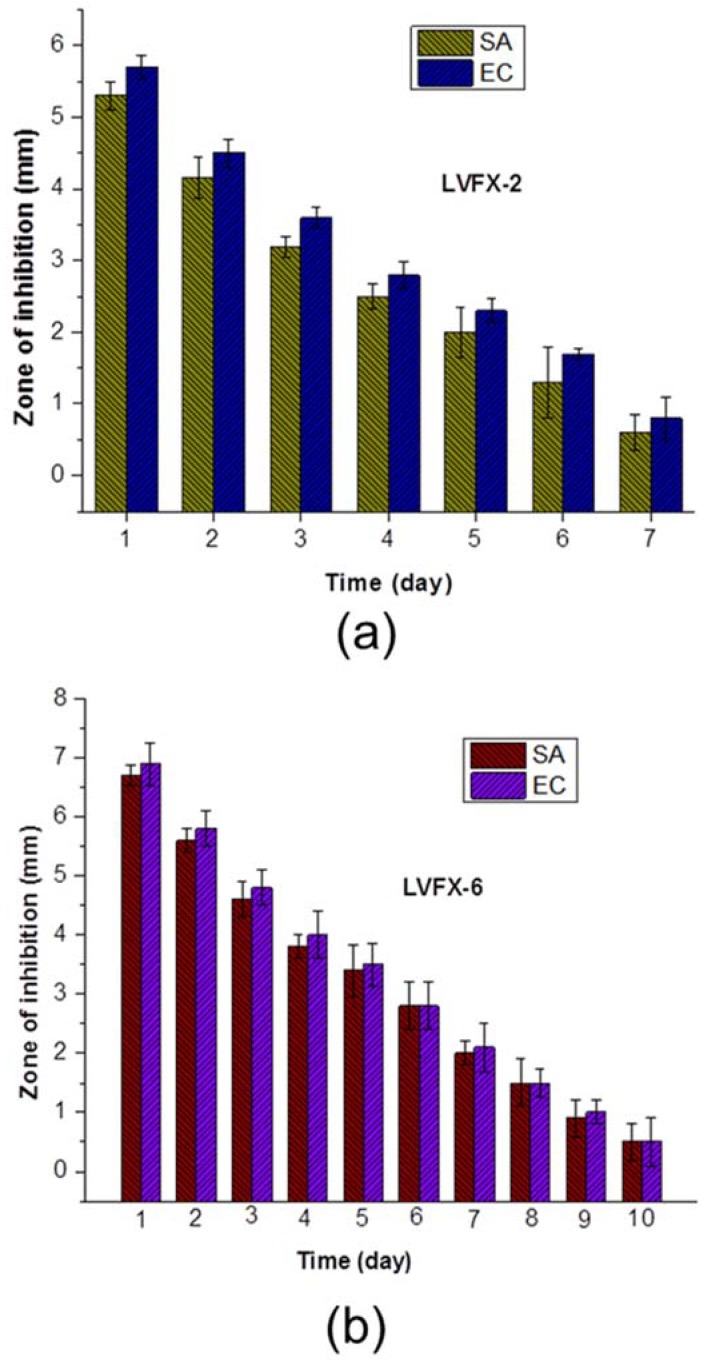
Sustained efficacy test performance (**a**) LVFX-2, and (**b**) LVFX-6. (*n* = 3).

Moreover, LVFX-2 and LVFX-6 (Figure 8) samples were further analyzed using sustained agar-diffusion tests to examine their durable antibacterial performance over the course of several days. These tests showed the antibacterial activity of the samples with the release of antibacterial agents after each 24-h period. LVFX-2 showed average inhibition zone and sustained-release antibiotic properties for 7 days against *E. coli* and *S. aureus*. However, LVFX-6 was more stable and showed an average inhibition zone with the release of antibacterial functions for 10 days against both types of bacteria. It can be observed (Figure 8a) that LVFX-2 showed slightly different inhibition zones for *S. aureus* and *E. coli.* During all 7 days, *E. coli* showed greater inhibition-zone diameter than *S. aureus*. Furthermore, the inhibition zone for both types of bacteria was reduced by more than 50% in first 3 days. The average minimum inhibition zone for *S. aureus* and *E. coli* was 0.6 mm and 0.8 mm, respectively. In the case of LVFX-6, the inhibition-zone diameter (Figure 8b) for both types of bacteria were almost similar and showed antibacterial properties for 10 days. Overall, the inhibition of *E. coli* by the meshes was always better than that of *S. aureus*. Interestingly, inhibition-zone diameters for the 6th, 8th, and 10th days for both bacteria were similar and inhibition-zone diameters gradually reduced for both types of bacteria (*S. aureus* and *E. coli*).

Previously, PP meshes have been surface modified with silver nanoclusters as an antimicrobial to prevent hernia-mesh infection [33]. Moreover, antibiotic-loaded polyester mesh has been tested using AATCC 100–2004 [34]. Since then, polyester mesh has been restricted in its use for hernia repair due to the formation of fistulae and other abdominal complications. Further, certain antibacterial methods may not be suitable for mesh-infection prevention. PP hernia mesh infection is a serious problem for hernia operations, and it may require antibacterial remedies for at least more than one week. Here, the CD-loaded antibiotics could be a good option for preventing mesh infection. 

Recently, this group has conducted the surface modification and coating of CD by incorporating triclosan as a model antibacterial agent and hydrophobic agent to simulate a slow-releasing pattern. Sustained antibacterial activity was achieved for up to 7 days [35]. Improvement of the process with a two-step grafting method of HDI and CD was able to improve the antibacterial activity to more than 10 days [29]. However, triclosan cannot be used as an antibacterial agent for hernia repair due to restrictions and growing environmental concerns for human safety. However, the antibiotic agent levofloxacin HCL has shown desirable antibacterial performance for 10 days. Thus, a PP surface modified implant with levofloxacin HCL as an antibiotic agent could be a suitable implant for hernia mesh-infection prevention.

### 4.5. Release Kinetics of Levofloxacin HCL 

**Figure 9:**
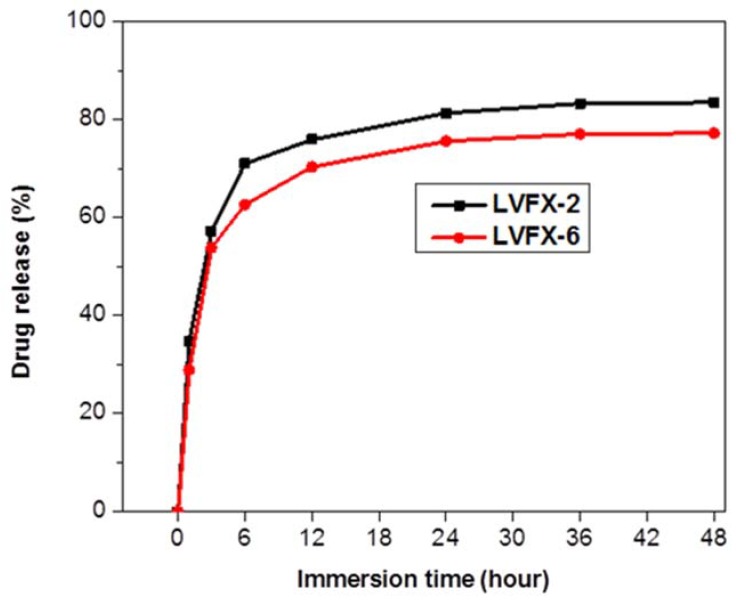
Drug release analysis of levofloxacin-loaded LVFX-2 and levofloxacin-loaded LVFX-6.

Figure 9 displays the slow release of levofloxacin-HCL-loaded samples LVFX-2 and CD-LVFX-6. It can be seen that both samples continuously released drugs for 48 h. Nevertheless, in first 6 h, a considerable amount of the levofloxacin HCL was released. However, the drug release was somewhat stable after 9 h, and after 12 h, the release was more stable. It can be seen that 12–48 h sustained release of drug was observed. However, LVFX-2 released 76% of the drug in first 12 h and a total 83.5% of drug was released in 48 h.

However, LVFX-6 released accumulative levofloxacin HCL of 70% in first 12 h and later up to 77% drug was released in 48 h. After 1 h, both samples of LVFX-2 and LVFX-6 released 34.6% and 28.8%, respectively, of the total drug amount, which was a greater amount than any hour during the first 48 h. This may be due to the fact that levofloxacin HCL was adhered to the surfaces of grafted CD and other inclusion complexes with CD. Therefore, CD-grafted samples displayed a burst release of levofloxacin HCL initially and showed sustained release after 12 h. Moreover, among both samples, LVFX-6 showed more stable and sustained drug release compared to LVFX-2. We also noticed the same trend of drug release for antibacterial properties. Therefore, it is confirmed that levofloxacin HCL is initially released in large amounts and then it reduces to a sustained release as time increases. 

Moreover, the burst release of antibiotics in the initial stage followed by sustained release could be an advantage for mesh-infection prevention due to the fact that after hernia repair, bacteria grow in the initial stage after surgery. Thus, such behavior of antibiotic release could be more suitable to cure mesh infection [36].

## 5. Conclusions

The CD-grafted PP meshes captured levofloxacin HCL and showed excellent antibacterial properties for 10 days. The low-pressure cold oxygen plasma effectively modified the inert PP meshes. HDI was successfully grafted onto oxygen–plasma-treated meshes. Moreover, CD was connected and formed covalent bonding with HDI and PP mesh fibers and loaded levofloxacin HCL. The modified PP mesh devices demonstrated suitable antibacterial properties and sustained drug-release kinetics. It was found that CD-grafted PP meshes loaded with levofloxacin demonstrated burst release of the drug during the first few hours but later showed sustained drug release. Thus, the yield product could be suitable for mesh-infection prevention.
